# A New Preoperative Risk Score for Predicting Postoperative Complications in Elderly Patients Undergoing Hepatectomy

**DOI:** 10.1007/s00268-021-05985-w

**Published:** 2021-02-17

**Authors:** Koichi Tomita, Itsuki Koganezawa, Masashi Nakagawa, Shigeto Ochiai, Takahiro Gunji, Kei Yokozuka, Yosuke Ozawa, Kosuke Hikita, Toshimichi Kobayashi, Toru Sano, Naokazu Chiba, Shigeyuki Kawachi

**Affiliations:** Department of Digestive and Transplantation Surgery, Tokyo Medical University Hachioji Medical Center, 1163 Tatemachi, Hachioji-shi, Tokyo, 193-0998 Japan

## Abstract

**Background:**

Postoperative complications are not rare in the elderly population after hepatectomy. However, predicting postoperative risk in elderly patients undergoing hepatectomy is not easy. We aimed to develop a new preoperative evaluation method to predict postoperative complications in patients above 65 years of age using biological impedance analysis (BIA).

**Methods:**

Clinical data of 59 consecutive patients (aged 65 years or older) who underwent hepatectomy at our institution between 2017 and 2020 were retrospectively analyzed. Risk factors for postoperative complications (Clavien-Dindo ≥ III) were evaluated using multivariate regression analysis. Additionally, a new preoperative risk score was developed for predicting postoperative complications.

**Results:**

Fifteen patients (25.4%) had postoperative complications, with biliary fistula being the most common complication. Abnormal skeletal muscle mass index from BIA and type of surgical procedure were found to be independent risk factors in the multivariate analysis. These two variables and preoperative serum albumin levels were used for developing the risk score. The postoperative complication rate was 0.0% with a risk score of ≤ 1 and 57.1% with a risk score of ≥ 4. The area under the receiver operating characteristic curve of the risk score was 0.810 (*p* = 0.001), which was better than that of other known surgical risk indexes.

**Conclusion:**

Decreased skeletal muscle and the type of surgical procedure for hepatectomy were independent risk factors for postoperative complications after elective hepatectomy in elderly patients. The new preoperative risk score is simple, easy to perform, and will help in the detection of high-risk elderly patients undergoing elective hepatectomy.

## Introduction

Generally, elderly patients have a high incidence of comorbidity (9.0–52.5%) and are usually considered a high-risk group for hepatectomy [1, 2]. According to a report of the Japanese Nationwide Survey on hepatocellular carcinoma (HCC) resection, elderly patients had significantly worse overall survival probabilities than younger patients (the 5-year overall survival probabilities: 68.8% vs. 59.5%; hazard ratio: 0.76). Furthermore, the cumulative incidence of HCC- or liver-related death was almost identical between elderly and younger patients, though the cumulative incidence of other causes of death was higher in the elderly (subdistribution hazard ratio: 0.32) [3]. For example, preoperative frailty in elderly patients undergoing hepatectomy is associated with age-related events such as cardiopulmonary complications, delirium, transfer to a rehabilitation facility, and dependency [4]. Thus, the indication of hepatectomy for elderly patients should be considered based on not only the tumor condition and liver function but also the risks specific to elderly patients.

However, predicting postoperative risk in elderly patients undergoing hepatectomy is not easy because of the lack of a reliable preoperative evaluation system. For example, estimation of physiological ability and surgical stress (E-PASS) [5] and physiological and operative severity score for the enumeration of mortality and morbidity (POSSUM) scores [6] have been reported previously for predicting postoperative complications in elderly patients with HCC. However, these systems are complicated and not specific for the elderly or hepatectomy. Simons et al. also reported an original risk score of in-hospital mortality for HCC resection [7]; however, this score cannot predict postoperative complications.

Therefore, we aimed to establish a new simple preoperative evaluation system for predicting postoperative complications in elderly patients undergoing elective hepatectomy. Biological impedance analysis (BIA), which is different from tests for the evaluation of tumor condition or liver function, was introduced in this study to evaluate patients’ preoperative body composition. BIA can be used to measure body water content, muscle mass, and fat mass using electrical impedance [8] and to diagnose decreased skeletal muscle, which is an element of sarcopenia and an important prognostic factor for hepatectomy for both HCC [9] and colorectal liver metastasis [10].

## Material and methods

### Subjects

**Fig. 1:**
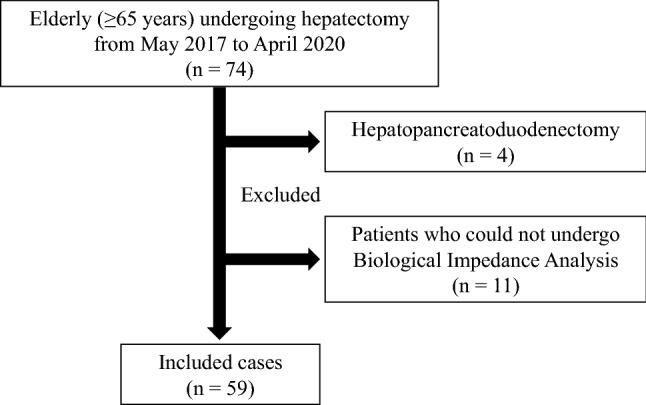
Patient enrollment flowchart

The clinical data of 74 consecutive patients (≥ 65 years) who underwent hepatectomy at the Tokyo Medical University Hachioji Medical Center between May 2017 and April 2020 were analyzed retrospectively. These included patients with HCC, liver metastasis, and gallbladder carcinoma. Patients undergoing surgical procedures ranging from partial hepatectomy to trisectionectomy were included; however, four patients undergoing hepatopancreatoduodenectomy were excluded because such patients are at a much higher risk for postoperative complications than patients undergoing other procedures included here [11]. Moreover, 11 patients who could not undergo BIA, for reasons described below, were excluded. After excluding these cases, the remaining 59 cases were included in this study (Fig. 1).

All patients underwent blood tests preoperatively, including serum total bilirubin, albumin, prothrombin, and indocyanine green tests. In terms of disease diagnosis, especially for patients with HCC, the pathological liver-fibrosis stage was evaluated according to the METAVIR fibrosis score [12].

This study was approved by the institutional review board of Tokyo Medical University (T2020-0174). Informed consent was obtained from all patients.

### BIA

In the present study, the InBody 770 (Biospace Co., Seoul, Korea) device was used for BIA. BIA was performed preoperatively in the physiology laboratory before or after admission. The InBody 770 device can be used to measure each patient’s characteristics in 90 s, with the patient holding the machine while standing upright. BIA can be used to measure body weight, water content (intra- and extra-cellular), amount of protein, skeletal muscle mass, muscle mass of extremities, fat mass, body cell mass, and minerals [8]. During the study, skeletal muscle mass (kg), skeletal muscle mass index (SMI), body fat (kg), and body cell mass (kg) were measured. The SMI cut-off value was based on the Asian Working Group for Sarcopenia (AWGS) criteria [13]. SMI values lower than 7.0 kg/m^2^ for male patients and 5.7 kg/m^2^ for female patients were considered abnormal. Generally, the BIA could not be performed in patients with metal objects inside the body, such as a cardiac pacemaker, or those who could not stand unassisted. These patients were excluded from this study.

### Evaluation of the liver function

At our institution, the preoperative evaluation of liver function is achieved by performing blood tests, including a liver function test, indocyanine green retention rate at 15 min, and ^99m^Tc-galactosyl human serum albumin (GSA) scintigraphy. The usefulness of ^99m^Tc-GSA scintigraphy was reported previously [14].

### Postoperative complications

The postoperative complications were graded using the Clavien-Dindo classification [15], which is a representative grading system for postoperative complications used worldwide. This study aimed to predict complications with a Clavien-Dindo Grade III or higher because such patients need invasive treatment.

### Development of new preoperative risk score

The risk factors for postoperative complications (Clavien-Dindo ≥ III) were evaluated by univariate and multivariate analyses. The risk score for predicting postoperative complications was developed based on significant factors and serum albumin from the multivariate analysis. Scores were assigned for each variable according to the beta-coefficient value from the logistic regression analysis.

### Surgical risk indexes

The new risk score was compared with other known surgical risk indexes. These included the American College of Surgeons National Surgical Quality Improvement Program (ACS NSQIP) surgical risk [16], American Society of Anesthesiologists (ASA) classification, E-PASS [17], POSSUM [18], and Portsmouth-POSSUM (P-POSSUM) [19].

### Statistical analyses and development of risk score

All statistical analyses were performed using IBM SPSS Statistics, version 26.0 (IBM Corp., Armonk, N.Y., the USA). Continuous variables are expressed as the median and range (minimum–maximum). The Mann–Whitney U-test was used to evaluate the significance of the difference between groups. Categorical variables were compared using the Chi-square test or Fisher’s exact test, as required. Multivariate analysis was performed using logistic regression analysis with a forward selection of the likelihood ratio. The performance of the risk score was evaluated using receiver operating characteristic (ROC) analysis, and the area under the ROC curve (AUROC) was calculated. All statistical tests were two-tailed, and significance was set at a *p*value of < 0.05.

## Results

### Patient characteristics

The patient characteristics are shown in Table 1. In total, there were 34 male and 25 female patients with a median age of 75 years. Hypertension was the most common comorbidity observed in patients. HCC and liver metastasis were diagnosed in 44.1% and 32.2% of the patients, respectively. The median SMI using the InBody 770 device was 7.1 kg/m^2^ in male and 5.8 kg/m^2^ in female patients; both were only slightly higher than the AWGS cut-off values. The number of patients who underwent the different surgical procedures was as follows: partial hepatectomy, 29 (49.2%); segmentectomy, 6 (10.2%); sectionectomy, 7 (11.9%); hemihepatectomy or bisectionectomy, 14 (23.7%); and trisectionectomy, 3 (5.1%). Combined biliary resection was performed in three cases (two for perihilar cholangiocarcinoma and one for gallbladder carcinoma).

**Table 1:** Patient characteristics

Factors				*n* = 59	
*General background*					
Age				years	75 (65–87)
Sex		Male			34 (57.6%)
		Female			25 (42.4%)
Comorbidity		Hypertension			38 (64.4%)
		Diabetes mellitus			22 (37.3%)
		Cardiovascular disease			9 (15.3%)
		Hyperlipidemia			6 (10.2%)
*Disease information*					
Diagnosis		Hepatocellular carcinoma			26 (44.1%)
			Etiology	HBV	4
				HCV	9
				Alcohol	6
				Others	7
			Fibrosis^a^	F0	2
				F1	5
				F2	5
				F3	7
				F4	7
		Liver metastasis			19 (32.2%)
		Gallbladder carcinoma			5 (8.5%)
		Intrahepatic or perihilar cholangiocarcinoma	3 (5.1%)		
		Liver cyst			2 (3.4%)
		Others			4 (6.8%)
*Preoperative evaluation*					
Blood test		Total bilirubin		mg/dL	0.6 (0.2–1.6)
		Albumin		g/dL	3.7 (2.8–4.3)
		Prothrombin		%	106 (77–148)
Child–Pugh classification		A			57 (96.6%)
		B			2 (3.4%)
Indocyanine green		R15			9.5 (2–32)
Biological impedance analysis		Body mass index		kg/m^2^	22.5 (16.6–29.2)
		Skeletal muscle mass index	Male	kg/m^2^	7.1 (5.7–8.9)
			Female	kg/m^2^	5.8 (4.3–6.9)
		Body fat		%	27.8 (13.4–40.7)
		Body cell mass		kg	25.7 (16.6–36.8)
ACS NSQIP surgical risk		Serious complication		%	12.0 (6.3–32.2)
ASA classification					2 (1–3)
E-PASS		Comprehensive risk score			9.99 (2.33–30.92)
POSSUM		Predicted morbidity		%	42.3 (18.5–98.8)
P-POSSUM		Predicted morbidity		%	38.5 (16.2–98.6)
*Surgical information*					
Procedure		Partial hepatectomy			29 (49.2%)
		Segmentectomy			6 (10.2%)
		Sectionectomy			7 (11.9%)
		Hemihepatectomy or bisectionectomy	14 (23.7%)		
		Trisectionectomy			3 (5.1%)
Combined biliary resection				3 (5.1%)	
*Surgical outcome*					
Operative time				min	260 (70–788)
Blood loss				mL	110 (0–1475)
Postoperative complication		Clavien-Dindo Grade	≤ II		47 (79.7%)
			III		10 (16.9%)
			IV		2 (3.4%)
Type of complication		Biliary fistula			8 (13.6%)
(Clavien-Dindo ≥ III)		Intra-abdominal abscess			2 (3.4%)
		Pulmonary complications			1 (1.7%)
		Ileus			1 (1.7%)
Postoperative stay				days	13 (6–197)

^a^Evaluated according to the METAVIR fibrosis score[12]

Continuous variables are expressed as median (minimum–maximum)

Categorical variables are expressed as the number of patients (%)

*ACS* American College of Surgeons; *ASA* American Society of Anesthesiologists; *E-PASS* estimation of physiological ability and surgical stress; *HBV* hepatitis B virus; *HCV* hepatitis C virus; *NSQIP* National Surgical Quality Improvement Program; *POSSUM* physiological and operative severity score for the enumeration of mortality and morbidity; *P-POSSUM* Portsmouth-POSSUM

In terms of the postoperative complications, 12 patients (20.3%) had Clavien-Dindo ≥ III, whereas no patient died (Clavien-Dindo V) during this study. Among the observed postoperative complications, biliary fistula was the most common (13.6%). There were few cases of other complications, such as intra-abdominal abscess, pulmonary complications, and ileus. None of these patients had posthepatectomy liver failure of Clavien-Dindo ≥ III.

### Univariate and multivariate analyses of postoperative complications

The results of the univariate and multivariate analyses of postoperative complications are shown in Table 2. In the univariate analysis, there was a significant difference in the serum albumin level (lower than 3.5 g/dL) and the type of surgical procedure between the two groups (Clavien-Dindo ≥ III and Clavien-Dindo ≤ II). Multivariate analysis revealed that abnormal SMI and the type of surgical procedure were independent risk factors for postoperative complications (*p* < 0.05).

**Table 2:** Logistic regression model for postoperative complications using univariate and multivariate analysis

Variables				Univariate analysis				Multivariate analysis	
				Clavien-Dindo ≤ II *n* = 47	Clavien-Dindo ≥ III *n*= 12	Odds Ratio (95% CI)	*P*value	Odds Ratio (95% CI)	*P*value
*General background*									
Age		Years		75 (65–87)	77 (67–82)	–	0.456		
		≥ 75		24 (51.1%)	8 (66.7%)	1.92 (0.51–7.24)	0.333		
Sex		Male		26 (55.3%)	8 (66.7%)	1.62 (0.43–0.61)	0.478		
Comorbidity		Hypertension		28 (59.6%)	10 (83.3%)	3.39 (0.67–17.25)	0.114	–	0.473
		Diabetes mellitus		18 (38.3%)	4 (33.3%)	0.81 (0.21–3.07)	0.514		
		Cardiovascular disease		6 (12.8%)	3 (25.0%)	2.28 (0.48–10.87)	0.261		
*Disease information*									
Diagnosis		Hepatocellular carcinoma		20 (42.6%)	6 (50.0%)	1.35 (0.38–4.81)	0.643		
*Preoperative evaluation*									
Blood test		Total bilirubin	mg/dL	0.6 (0.2––1.6)	0.55 (0.3–0.8)	–	0.530		
		Albumin	g/dL	3.8 (3.1–4.3)	3.5 (2.8–4.2)	–	0.364		
			≤ 3.5	12 (25.5%)	7 (58.3%)	4.08 (1.09–15.31)	0.037	–	0.067
		Prothrombin	%	106 (77–148)	106 (84–126)		0.895		
Child–Pugh classification		B		2 (4.3%)	0 (0.0%)	–	0.632		
Indocyanine green		R15		9 (2–32)	10 (3–16)		0.778		
InBody 770		Body mass index	kg/m^2^	23.1 (16.6–27.8)	21.7 (19.0–29.2)	–	0.175	–	0.873
		Skeletal muscle mass index	Abnormal	18 (38.3%)	8 (66.7%)	3.22 (0.85–12.26)	0.077	7.03 (1.31–37.62)	0.023
		Body fat	%	15.5 (5.7–25.0)	14.1 (11.6–27.1)	–	0.985		
		Body cell mass	kg	25.7 (17.3–36.8)	25.6 (16.6–34.9)	–	0.799		
*Surgical information*									
Procedure		Partial or segmentectomy		31 (66.0%)	4 (33.3%)	–	0.048	3.40 (1.39–8.30)	0.007
		Sectionectomy		6 (12.8%)	1 (8.3%)				
		Bisectionectomy or more		10 (21.3%)	7 (58.3%)				

Continuous variables are expressed as medians (minimum–maximum)

Categorical variables are expressed as the number of patients (%)

Abnormal skeletal muscle mass index was diagnosed based on the definition of the Asian Working Group for Sarcopenia

*CI* confidence interval

### Risk score for predicting postoperative complications

**Fig. 2:**
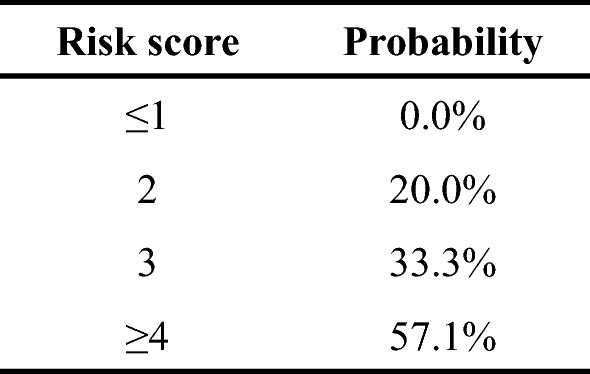
Preoperative risk score and the probability of postoperative complications

Based on the results of the multivariate analysis, abnormal albumin levels, abnormal SMI, and the type of surgical procedure were used for developing the risk score for predicting the postoperative complications. The scores were assigned based on the coefficient value for each item in the logistic regression model (Table 3). The risk score of patients ranged from 0 to 5. The estimated probability of postoperative complications between each risk score is shown in Fig. 2. The postoperative complication rate was 0.0% for patients with a risk score of ≤ 1 and 57.1% for those with a risk score of ≥ 4.

**Table 3:** Preoperative risk score for postoperative complications

Parameter	Score		
	0	1	2
Serum albumin (g/dL)	> 3.5	≤ 3.5	
Skeletal muscle mass index	Normal		Abnormal
Type of surgical procedure	Partial or segmentectomy	Sectionectomy	Bisectionectomy or more

### Comparison of the risk score performance

**Fig. 3:**
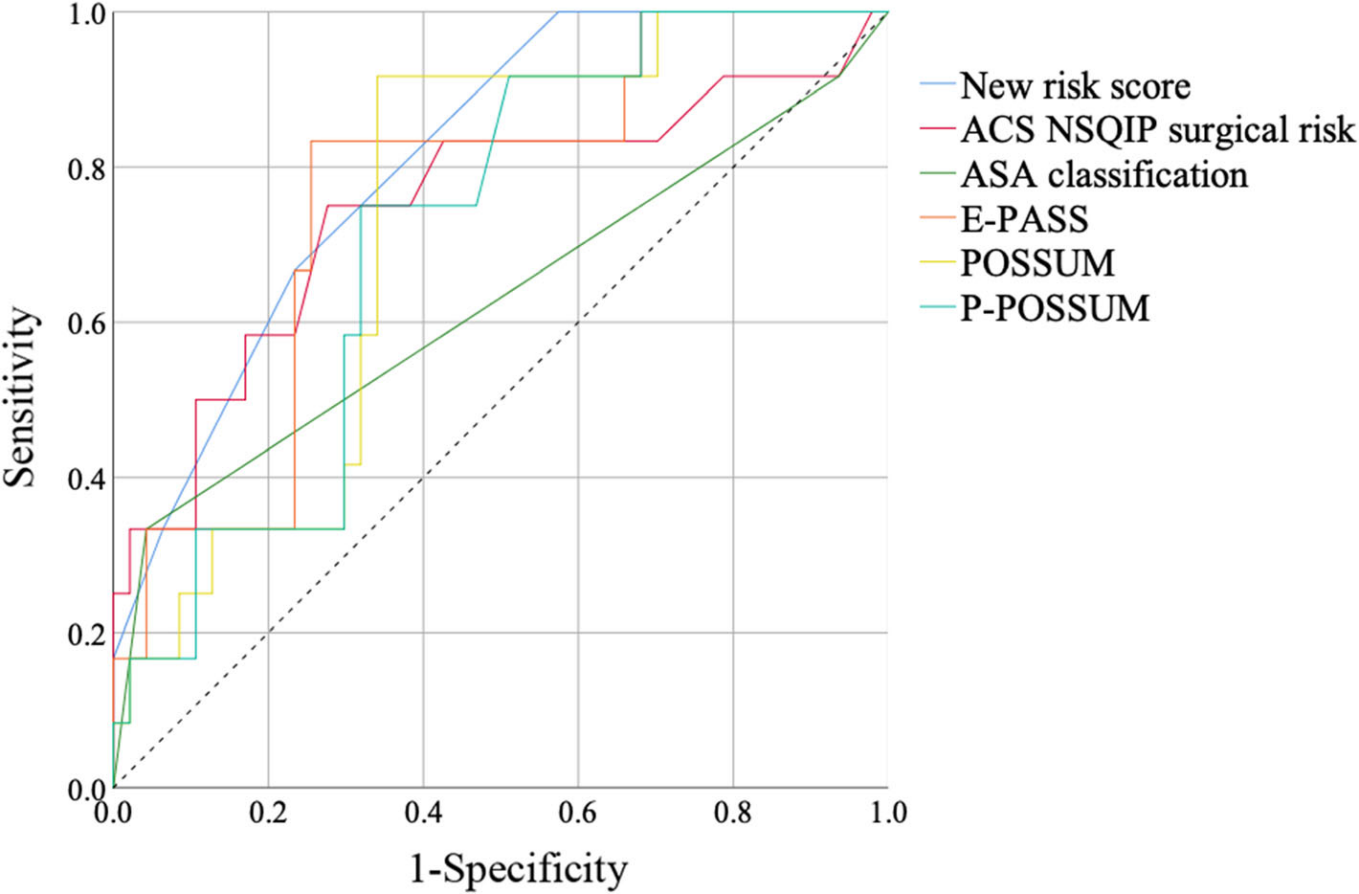
Receiver operating characteristic (ROC) analysis of each risk score for predicting postoperative complications (Clavien-Dindo ≥ III) Dashed line: reference

The results of the ROC analysis of the new risk score and other known surgical risk indexes for predicting postoperative complications are shown in Fig. 3. The corresponding AUROCs are shown in Table 4. Among these scores or indexes, the new risk score showed the best result, with an AUROC of 0.810 (*p* = 0.001).

**Table 4:** Receiver operating characteristic analysis of surgical risk indexes in predicting the postoperative complications

	AUROC	95% CI	*p*value
New risk score	0.810	0.689–0.932	0.001
ACS NSQIP surgical risk	0.748	0.570–0.926	0.008
ASA classification	0.627	0.423–0.831	0.178
E-PASS	0.761	0.615–0.907	0.006
POSSUM	0.730	0.591–0.870	0.014
P-POSSUM	0.715	0.571–0.858	0.023

*AUROC* area under the receiver operating characteristic curve; *CI* confidence interval

## Discussion

The present study revealed that preoperative decreased skeletal muscle and the type of surgical procedure used for hepatectomy appeared to have a statistically significant impact on the Clavien-Dindo ≥ III postoperative complications among patients aged 65 years or older undergoing hepatectomy. In this report, the preoperative risk assessment of the elderly undergoing hepatectomy is discussed from a new perspective using BIA.

Recently, sarcopenia has increasingly been recognized as an important factor in predicting postoperative complications and long-term prognosis among patients undergoing gastrointestinal surgery [20]. Decreased skeletal muscle is an important element of sarcopenia. The guidelines from the American College of Surgeons have also highlighted the importance of assessing sarcopenia before surgical oncology in the elderly [21]. Valero et al. showed that a low preoperative psoas muscle mass is an independent risk factor for postoperative complications in patients with Clavien-Dindo ≥ III undergoing hepatectomy and liver transplantation for HCC [22]. Higashi et al. similarly reported that sarcopenia is a risk factor for postoperative complications in patients undergoing major hepatectomy. They also mentioned that sarcopenia is a risk factor for liver-related morbidity and mortality in patients aged > 70 years [23]. According to another report, low skeletal muscle mass and quality are also related to mortality after resection of intrahepatic cholangiocarcinomas [24]. Thus, sarcopenia or decreased skeletal muscle is a risk factor for hepatectomy, though it has not been used as an item of preoperative risk index before.

In the present study, we used the BIA for diagnosis of decreased skeletal muscle mass. BIA can be used to evaluate the body composition precisely by calculating the electrical impedance of a patient’s body. The usefulness of the BIA has already been confirmed widely in liver cirrhosis [25] and living donor liver transplantation patients [26]. However, in almost all of the previously cited reports, the psoas or skeletal muscle mass was calculated either using L3-level computed tomography (CT) or magnetic resonance imaging (MRI). The area of skeletal muscle at the L3 level was directly correlated with whole-body skeletal muscle [27]. This method had the advantage that most of our hepatectomy patients underwent CT or MRI preoperatively, meaning that there was no need for performing other tests. These methods approximate the BIA, though they can be used as a substitute for BIA, especially when an institution lacks BIA equipment. However, the BIA has the disadvantage that the optimal cut-off values for SMI in the elderly population have not been determined. Because of the lack of optimal cut-off values of SMI in both the elderly population and in hepatectomy patients, the AWGS criteria were used in this study. Further studies will be needed to determine the optimal cut-off value, especially in elderly patients.

Furthermore, there are some reports on the relationship between body composition (other than muscle mass) and postoperative complications in hepatectomy. Low preoperative body cell mass is a risk factor for infections associated with mortality in cases of living donor liver transplantation [26]. In line with this study, we evaluated the body cell mass, though no significant difference was observed in our study cohort. Hamaguchi et al. reported that preoperative muscle steatosis in patients undergoing hepatectomy for HCC is an independent risk factor for postoperative complications in patients with Clavien-Dindo ≥ III and infectious complications [28]. In another study, it was reported that a low preoperative SMI value, high intramuscular adipose tissue content, and high visceral-to-subcutaneous adipose tissue-area ratio were risk factors for mortality and recurrence in patients undergoing hepatectomy for HCC [29]. In this study, only total body fat was evaluated, with no significant difference observed in this parameter.

Serum albumin was previously shown to be an independent risk factor for postoperative complications in elderly patients undergoing hepatectomy [5]. Therefore, the preoperative serum albumin levels, in addition to the SMI value and the type of surgical procedure, were also included in the development of the new preoperative risk score for predicting postoperative complications. Based on these three items, we developed a new, simple preoperative risk score for elderly patients who underwent elective hepatectomy. There are already some other established risk assessment systems. For example, the ACS NSQIP surgical risk [16], E-PASS [17], POSSUM [18], and P-POSSUM [19] also showed relatively good results with significant differences in ROC analysis for predicting postoperative complications. However, our risk score showed better results and may be simpler and easier to use than these other risk assessment systems. Additionally, our risk score may have better performance than other systems because it focuses on hepatectomy in elderly patients and uses BIA, which has not been used in other systems.

Despite these significant findings, the present study has several limitations. This study was performed in a single center with a relatively small number of patients. Therefore, our risk score should be validated in other subjects in a multicenter study. Further, patients in whom BIA could not be performed, such as those with metal objects inside the body, or those who could not stand unassisted, were excluded from the study. Therefore, our risk score may not be applicable directly for these patients. Evaluation of psoas muscle mass at the L3 level is a possible substitute for BIA, although further studies are needed for confirmation. Also, elderly patients who were deemed too frail for the surgery did not undergo hepatectomy. Therefore, further prospective studies will be needed to exclude this preselection bias.

## Conclusion

In conclusion, the present study revealed that decreased skeletal muscle and the type of surgical procedure for hepatectomy are independent risk factors for postoperative complications after elective hepatectomy in elderly patients. Furthermore, the new preoperative risk assessment system developed using serum albumin levels, SMI, and surgical procedure will help in the identification of high-risk elderly patients undergoing elective hepatectomy.
